# Degradation of Glaukonite Sandstone as a Result of Alkali-Silica Reactions in Cement Mortar

**DOI:** 10.3390/ma11060924

**Published:** 2018-05-30

**Authors:** Przemysław Czapik

**Affiliations:** Department of Building Engineering Technologies and Organization, Kielce University of Technology, Al. Tysiąclecia Państwa Polskiego 7, 25-314 Kielce, Poland; p.czapik@tu.kielce.pl; Tel.: +48-41-34-24-596

**Keywords:** alkali-silica reaction, microstructures, sandstone degradation, petrography, SEM-EDS

## Abstract

The mechanism of concrete degradation as a result of an alkali-silica reaction (ASR) largely depends on the mineral composition and microstructure of the reactive aggregate. This paper shows the reactivity results of quartz-glaukonitic sandstone, which is mainly responsible for the reactivity of some post-glacial gravels, available in Poland. After initial petrographic observations under a light microscope, the mode of sandstone degradation triggered by the reaction with sodium and potassium hydroxides was identified using scanning electron microscopy (SEM). It has been found that chalcedony agglomerates present in sandstone are separated from the rock matrix and subsequently cause the cracks formation in this matrix. Additionally, microcrystalline and potentially reactive silica is also dispersed in sandstone cement.

## 1. Introduction

The degradation of concrete caused by alkalis is usually associated with the presence of aggregate containing reactive forms of silica. This is so called alkali-silica reaction (ASR). Next to the alkali-carbonate reaction (ACR), this reaction is one of the deleterious alkali-aggregate reaction (AAR) variants, which are caused by the reaction of sodium and potassium hydroxides solutions with reactive aggregates [1]. However, the initial mechanism of concrete destruction for both cases is quite similar [1,2,3,4,5,6]. The reactive silica is depolymerized by hydration and the unbalanced charges at the surface of the dissolved silica particles are neutralized. After that, the hydrogen bond O–H at the silica surfaces is left with a weakly acidic character. In a strongly alkaline solution the hydrogen cations H^+^ at the silica surface can be replaced by the cations Na^+^ or K^+^ [1]. The main difference between various types of reactive aggregates is the rate of their reaction with alkalis occurring in concrete pore solutions [7]. 

There are some factors affecting the reaction of various aggregates with alkalis which should be taken into account. First of all is the availability of the alkalis [7,8], and the form of reactive silica occurring in the aggregate [9]. The texture of rock is the other significant factor [6,10,11,12].

Sodium and potassium hydroxides, reacting with aggregate in concrete, are mainly introduced with cement. As a result of dissolution of sodium and potassium sulphates from cement and the hydration of clinker phases which are alkali incorporating solid solutions, the alkalis are transferred to a concrete pore solution [1,2,3,7]. The other concrete components, such as chemical admixtures [13], mineral additions or even the aggregate, can also be a source of active alkalis [11,14,15]. However, not all alkalis present in concrete components are released to the solution soon after mixing with water.

Silica reactivity is known for its solubility. Because of this, the reactivity of various silica forms increases in the following sequence: quartz < tridymite < cristobalite < chalcedony, moganite < amorphous silica (opal, volcanic glass, synthetic glass) [9,16]. 

Besides the two aforementioned factors, this means the availability of alkalis and silica reactivity during the alkali-aggregate reaction and the texture of the aggregate may also have a significant influence [5,10,11,12]. The texture can reveal, for example, some specific distribution of reactive and unreactive phases in aggregate. The case in which the whole aggregate particle contains only one reactive mineral phase is very rare in nature [17,18]. However, those cases are usually considered in model tests [19,20,21,22,23,24]. Synthetic borosilicate glass, which is used in tests according to the ASTM C441/C441M Standard [25], belongs to those types of aggregates. The aggregate containing few reactive phases is also used in model tests, as in the case of opal, which can be composed of an amorphous phase (opal-AN), cristobalite and tridymite (opal-CT) [26]. Reactivity of these phases is different, therefore the reaction with alkalis on aggregate surface can occur only in some spots.

The polymineralic aggregate is usually used for concrete production. Besides the reactive phase, its particles also contain unreactive phases. Sometimes, one aggregate particle can contain more than one reactive phase. Although the unreactive phases do not react with alkalis, they can influence the reaction. Their main role consists in the limitation of alkalis access to reactive phases and preventing the expansion of reaction products. Due to the mutual location of reactive and unreactive phases, the progress of particular aggregate grains degradation can be different [12,27]. In the case of the same reactive phase, it can be manifested by different stress values and the different nature of cracks involved.

Other than the reactive silica and unreactive phases, the aggregate particles can contain the other phases, the presence of which can influence an alkali-silica reaction in concrete. Dolomite, which during the dedolomitization reaction can facilitate the access of alkaline solution to silica contained therein, can be assigned to these other phases [3,4,5].

The rock porosity is also associated with their texture. The “transport facility” of alkalis depends on the pores size, shape and distribution. Alkaline silica gel can also accumulate inside the larger pores of aggregate [3,12,28,29,30]. 

The degradation process of concrete will proceed differently, due to the aforementioned variations in the particles structure of reactive aggregate. Therefore, the degradation process of the aggregate [12] and also its surroundings (hardened cement paste [31]) can be distinguished. 

The aforementioned problem of texture in relation to the alkali-silica reaction is significant in the case of polymineralic aggregates, such as sandstones from the group of sedimentary rocks. 

In the presented work the results of the studies intend to determine how a sodium and potassium reaction with selected quartz-glaukonitic sandstone containing clay-carbonate binder is reported. Previously, the studies [32] indicated that this sandstone was the main component of post-glacial gravel, responsible for its reactivity. This gravel is one of the few examples of reactive aggregates occurring in Poland [32,33,34,35]. 

In this paper the details relating to the quartz-glaukonitic sandstone and subsequently to the concrete degradation as a result of an ASR reaction are reported. The studies were conducted on sandstone and also on degraded mortar.

## 2. Materials and Methods 

### 2.1. Sample Preparation

Pleistocene post-glacial gravel from northern Poland was used in the studies. It was obtained from a gravel pit as an aggregate fraction 8–16 mm. The postglacial gravel composition, with the highlighted of its reactive components, is show in Table 1.

Sandstone reactivity in cement paste was assessed on the basis of two mortar expansion tests [36,37]. Portland cement CEM I 42.5R, crushed gravel aggregate was used to prepare these mortar. Gravel aggregate was crushed mechanically to the fraction 0.125–4 mm. The chemical composition of cement is shown in Table 2. Additionally, the microstructure of the degraded cement-zeolite mortar containing this gravel was tested. Its degradation was manifested by the leaching of alkali silica gel on the surface, despite the presence of natural pozzolan (zeolite) [38,39].

### 2.2. Alkali Reactivity Testing

Reactivity of this gravel was determined according to the methodology of ASTM C1260 [36], ASTM C227 [37] and PN-92/B-06714-46 [40] Standards. 

In the test according to ASTM C 1260, the samples are stored immersed in 1 mL NaOH solution, in temperature = 80 °C. Expansion study was conducted for 14 days.

In the ASTM C 227 [37] study, the samples are stored temperature = 38 °C, moisture = 100%. Expansion study was conducted for 360 days.

Measurements of linear changes of samples were carried out with the Graff-Kaufman apparatus, with accuracy 0.005 mm. The results were presented as the average of 5 specimens measurements.

In these experiments the two mortars in which the reaction of alkali with reactive sandstone has undergone in different conditions have been obtained. The specimens obtained from these tests have been subjected to the further studies, with aim to explain the glaukonite sandstone degradation mechanism.

In the PN-92/B-06714-46 [40] study, reactivity was determined by measuring the loss of mass of aggregate immersed in a 10% solution of NaOH for 1 h, at a temperature of 90 °C. The test was carried out on 1 kg samples of 8–16 mm fractions. Prior to testing, the aggregate was rinsed with water to remove fine fractions and dried to constant weight at 105 °C to the nearest 0.1 g. After removed from the NaOH solution, the aggregate was washed under a stream of water until the removed effect of discoloration of used water after addition of phenolphthalein. The washed aggregate was dried to constant weight at 105 °C and weighed to the nearest 0.1 g.

Particles of quartz-glaukonitic sandstone separated from gravel aggregate were also examined by the same method. The results were presented as the average of two measurements.

### 2.3. Physical Properties of Sandstone 

The open water porosity and apparent density of quartz-glaukonite sandstone were determined by hydrostatic method operating on the basis of Archimedes’ principle [41]. It consists of comparing the mass of the sample measured in the dry state after immersing in water (so that the open pores are filled with water) and in the moist state.

### 2.4. Microscopic Observations

The mineral composition of sandstone was determined through observations of standard polished sections (35 × 25 mm) in transmitted light using a polarizing microscope BX51 (Olympus, Tokyo, Japan) equipped with digital camera. The thin sections were prepared without the use of cooling agents that could rinse the alkali-aggregate reaction products. The quantitative analysis was conducted by the point method using the integration table. Microscopic analyses included studies of mineral and particle size composition. About 1000 mineral grains in each sample were counted. The analyses were carried out in microscopic formulations for polarized passing light using a semiautomatic meter.

Afterwards, these studies were complemented by the analysis of polished sections surface carried out under the scanning electron microscope (SEM; FEI Company, Hillsboro, OR, USA). The SEM was also used to observe the microstructure of mortar. A degraded cement-zeolite mortar containing crushed gravel was selected for the tests. This analysis focused on identifying the quartz-glaukonite sandstone damages which occurred in the cement paste environment. The morphology of the resulting alkali-silica reaction products and the distribution of elements in sandstone undergoing reaction were determined. These tests were performed in environmental scanning electron microscope Quanta FEG 250 (FEI Company, Hillsboro, OR, USA) equipped with EDAX X-ray microanalyzer (EDS). Non-sputtered samples were tested under high-vacuum condition (pressure < 0.01 Pa). The images in the detector SE were obtained through the scanning of sample surface by the electron beams at voltage of 5 kV.

## 3. Results

### 3.1. Identification of Reactive Phases

**Figure 1:**
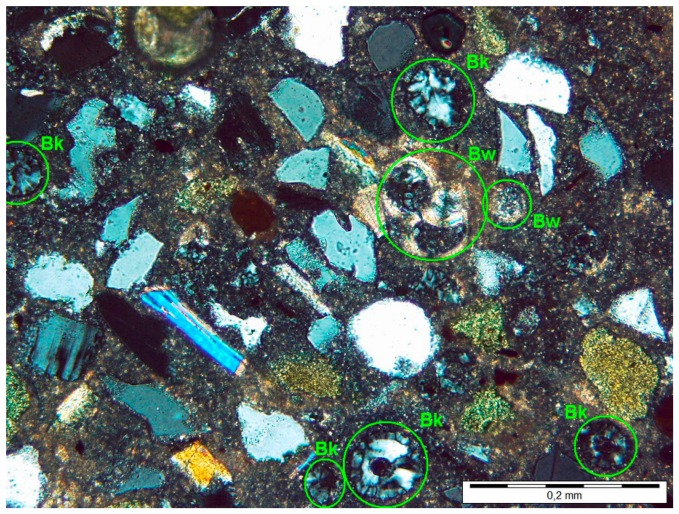
Texture of quartz-glaukonitic sandstone with clay-carbonate cement at 120× magnification. Marked silicate (Bk) and carbonate (Bw) bioclasts.

Quartz-glaukonitic sandstone surface with clay-carbonate cementing substance obtained during the observations under the light microscope is shown in Figure 1. This surface has the form of loose particle framework built from quartz grains, glaukonite aggregates, as well as silicate (Bk) and carbonate (Bw) bioclasts. These grains of size 15–100 μm are embedded in cementing substance build from clay minerals, microcrystalline silica, microsparitic calcite and organic material.

Silicate bioclasts in the form of sponge needles are built from fibrous chalcedony. They occur in two cross sections: longitudinal and transverse, and their maximum dimension reaches 280 μm. In both cases, the empty central channel is often visible. The needles length reaches 0.4 mm with diameter not exceeding 0.1 mm.

Fibrous chalcedony sometimes occurs also inside the carbonate bioclasts represented by shells of multichamber calcareous foraminifera. Size of this type of bioclasts varies from 0.1 to 0.2 mm.

Results of quantitative planimetric analysis of examined rock are shown in Table 3. 

**Figure 2:**
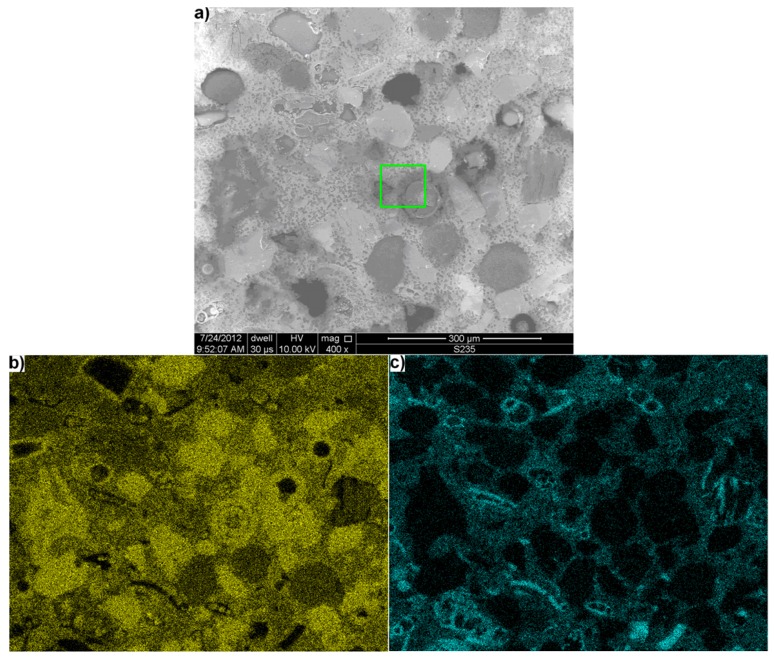
Microstructure of quartz-glaukonitic sandstone with clay-carbonate cement: (**a**) an image at 400× magnification and distribution on its surface (In the green rectangle, is marked the area selected for further analysis on Figure 3), (**b**) Si; and (**c**) Ca.

The image of polished section surface of quartz-glaukonite sandstone obtained under the scanning electron microscope is shown in Figure 2a. The psammite microstructure of sandstone, in which numerous grains with size about 100 μm are bonded by inhomogeneous cementing substance, is shown in this figure too.

Figure 2b,c show the distribution of silicon and calcium in the form of mapping obtained using the X-ray microanalyzer. It was found that silicon (Figure 2b), besides its mainly occurrence in quartz grains composition, is also present in significant amount in the binder bonding aggregate grains. Silica shown in Figure 4b can also occur in a form similar to amorphous silica fume, forming the spherical grains with diameter from 25 to 70 μm. They can be silicate bioclasts from the analysis under the light microscope.

Calcium (Figure 2c) occurs almost exclusively in cement, forming the envelopes with higher concentration around some grains rich in silicon. They can be carbonate bioclasts. On the image taken with the SE (Secondary Electron) detector (Figure 2a), these bioclasts are not as clearly visible as the silicate bioclasts. It also forms the individual concentrations in these grains, in which the presence of silicon is not detected. Part of them can be identified as residues of living organisms—foraminifera.

**Figure 3:**
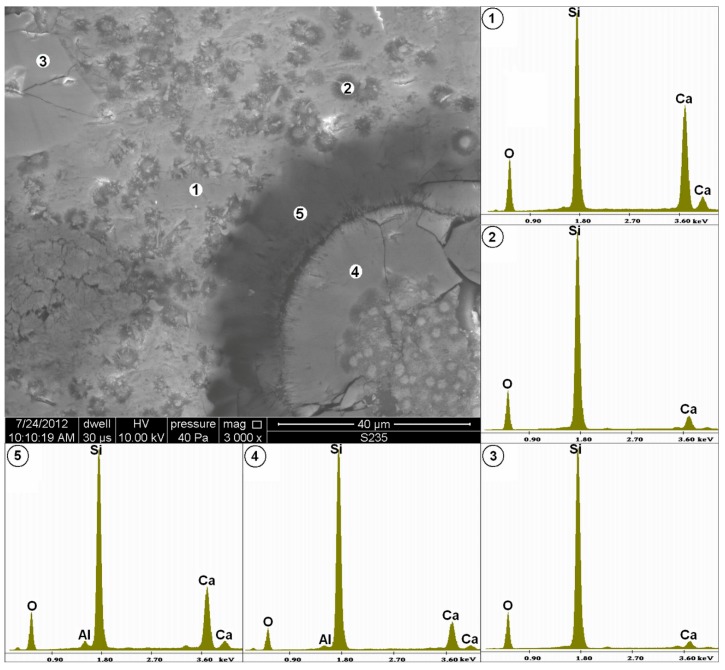
Microstructure of quartz-glaukonitic sandstone with clay-carbonate cement (area marked in Figure 2a), the image at 3000× magnification and with X-ray microanalysis.

While analyzing bonding substance in sandstone, one can detect that it is inhomogeneous (Figure 3). The point X-ray microanalysis, in point ①, reveals that this material is mainly composed of silicon and calcium. It is built of numerous small grains with size not exceeding 5 μm. They are mainly composed of silicon ②, which occur in the form of large quartz grains ③ bonded by “cement”. Thus, they can be regarded as micro and cryptocrystalline quartz and chalcedony, classified as reactive components.

In the bottom right corner of Figure 3, one of the aforementioned spherical grains of bioclast is visible. The X-ray microanalysis of its surface ④ shows that it is mainly composed of silicon with a small addition of calcium. Its external surface has a visibly cracked and defected layer. 

The occurrence of the modified bonding material zone with lower calcium content was identified around the studied spherical silicon rich agglomeration ⑤. 

### 3.2. Gravel and Sandstone Reactivity

**Figure 4:**
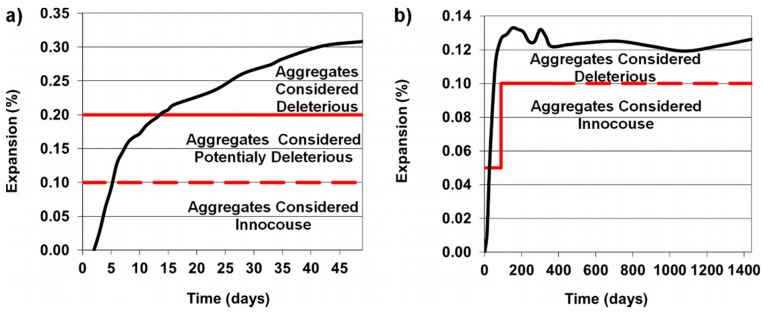
Expansion of mortar with gravel aggregate according to (**a**) ASTM C 1260, (**b**) ASTM C 227.

Figure 4 shows the results of the mortar expansion caused by the alkali-silica reaction. In both tests, the achieved expansion indicates that the used aggregate has reactivity that can result the concrete degradation.

**Figure 5:**
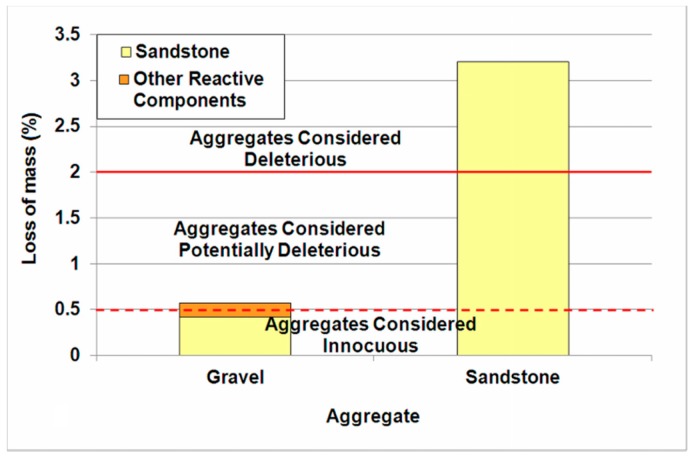
Loss of mass for particular aggregate fractions in NaOH solution.

Chemical testing of the post-glacial gravel aggregate confirmed its potential reactivity (Figure 5, left column). The quartz-glaukonitic sandstone is mainly responsible for this effect. The sandstone in 8–16 mm gravel, stored in NaOH solution causes the loss of mass lower than 0.5%, and is thus insufficient to classify this natural aggregate as potentially reactive. However, it can be classified as reactive because of the other reactive components [33].

The loss of mass determined during measurement of sandstone particles, selected from the gravel (Figure 5, right column) was 3.21%, so it exceeded the standard limit of 2%, thus it can be stated that it is a reactive rock.

After the measurement of sandstone reactivity, the occurrence of fine rock powder was found at the bottom of the beaker, filled with a NaOH solution.

### 3.3. Physical Properties

The average open porosity and bulk density of quartz-glaukonitic sandstone is shown in Table 4. High sandstone porosity, which is associated with its low density, testifies to the fact that alkaline solutions can easily migrate from its surroundings to the center of a specimen and to the reactive minerals. The surface of pores can also the increase of potential sites, on which the alkali-aggregate reaction can occur. 

### 3.4. Sandstone Degradation in Mortar 

During the observations under the scanning electron microscope of cement-zeolite mortar a number of areas with damaged microstructures and with products of alkali-silica reactions were found. 

**Figure 6:**
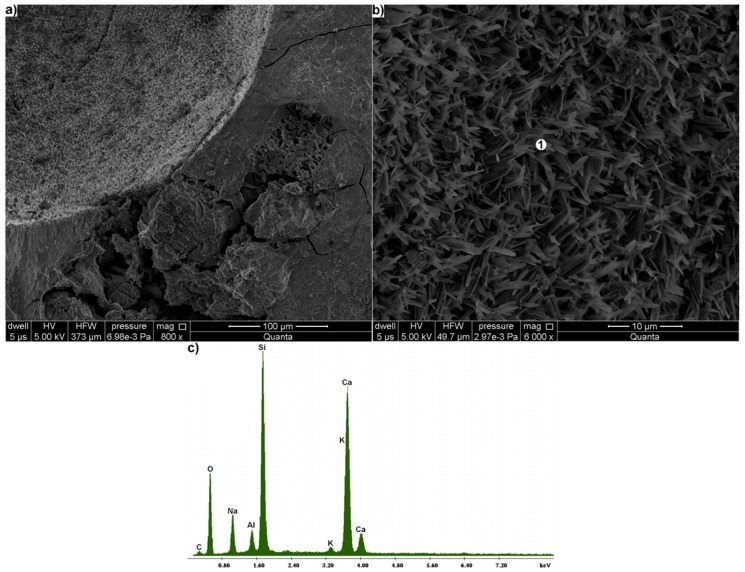
Microstructure of mortar: (**a**) pore in mortar at 800× magnification; (**b**) products of alkali-silica reaction, filling it; (**c**) X-ray microanalysis in point 1.

Products of alkali-aggregate reaction often occurred in pores. Having a place that can be filled, the gel in the pores takes the crystalline form (Figure 6a,b) [42]. Besides high content of silica and calcium, it is also characterized by increased sodium content (Figure 6c).

**Figure 7:**
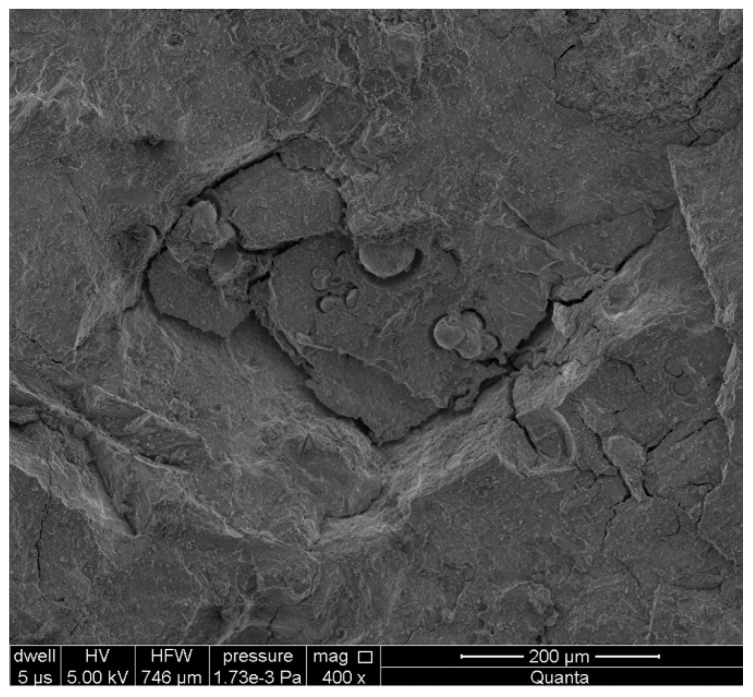
The cracked aggregate (in the central part of the figure) in the cement matrix.

**Figure 8:**
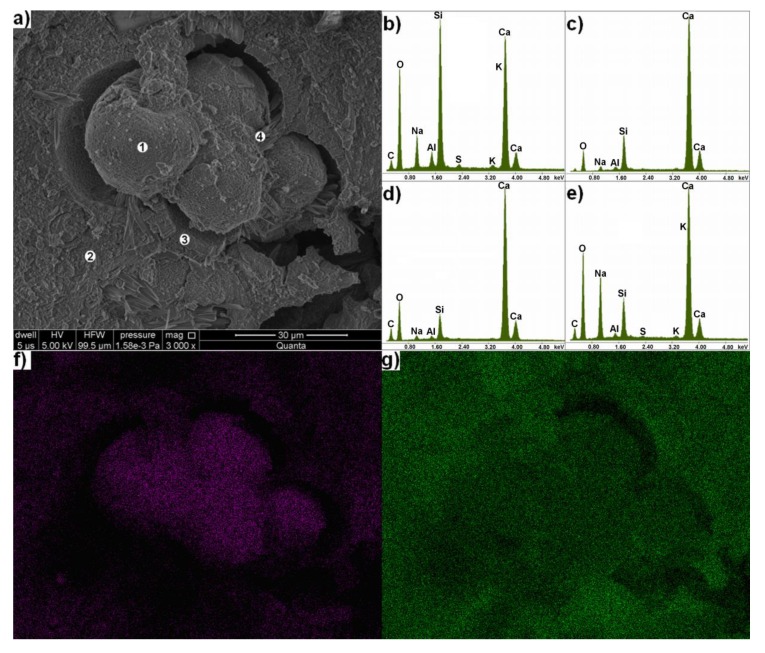
(**a**) a cracked grain of carbonate aggregate in mortar at 3000× magnification and X-ray microanalysis of elemental composition in point; (**b**) 1; (**c**) 2; (**d**) 3; (**e**) 4, and distribution maps of (**f**) Si; and (**g**) Ca.

There is also the occurrence of damaged aggregate particles, for whose degradation the alkali-silica reaction can also be considered (Figure 7 and Figure 8a). These particles were partly separated from paste by crack propagating along the paste-aggregate interface. The other cracks propagation was observed from this crack into the paste. The additional cracks also occurred in aggregate. They had an oval shape, which caused the separation of some spherical forms from the aggregate cement material. These forms have size from 6 to 60 μm.

The X-ray microanalysis reveal that separating spheres are mainly composed of silicon and calcium, with significant sodium content (Figure 8b). It was also found that they contain much less calcium compared to the surrounding aggregate cement (Figure 8c). Besides calcium, small silicon content was found in the cement.

The occurrence of a layer built of columns, perpendicularly growing out from cementing material and crack was found between this cement and spherical silicon agglomerations. The elemental composition of this layer is similar to cement composition (Figure 8d).

Acicular forms rich in calcium and sodium occur in the crack between spherical silicon agglomerations and layer rich in calcium (Figure 8d). This is the same phase, which, as a product of alkali-silica reaction, fills the pores of this mortar (Figure 5). 

The mapping of silicon and calcium distribution using the X-ray microanalyzer was carried out because of variable contents of these elements in damaged aggregate particle. These data confirmed the results of point X-ray microanalysis. Silicon content is significantly greater in separating spherical forms (Figure 7f). However, calcium is accumulated in cementing material, and increase of its concentration occurs in layer surrounding silica agglomerations (Figure 8g). This layer is also poorer in silicon compared to the rest of the material. 

**Figure 9:**
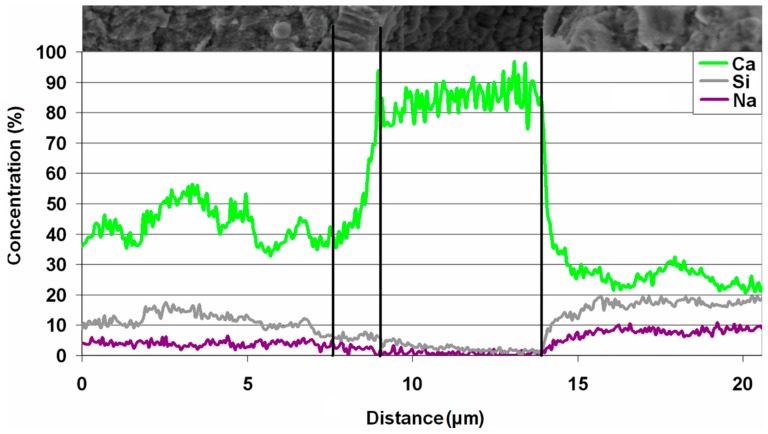
Changes of elements concentrations between carbonate cement and spherical silicon agglomeration from Figure 8a.

The elements distribution in the contact zone between cement and spherical silicon agglomeration was additionally determined using linear mapping shown on Figure 9. It presents the change of elements contents, on the way from aggregate cementing material to the center of the separating spherical form. In its consequence, it was stated that changes in element concentrations primarily concern calcium, silicon and sodium. Some fluctuations of their concentrations occur in the inhomogeneous cementing material. However, the role of calcium is dominant. Its content significantly increases in the layer formed on cementing material, separated from spherical silicon agglomeration by the 5 μm crack. This envelope thickness is 1.5 μm. Its surface is mainly composed of calcium. Silicon and sodium are almost undetectable in this envelope and they can occur only in trace amounts. Silicon and sodium amounts are significantly higher on the surface of separating spherical form, compared to aggregate cementing material, and calcium content is lower.

## 4. Discussion

These studies confirmed the sandstone reactivity, the reactive phases contained in it were identified and the progress of its degradation in the cement mortar was described.

The results of the chemical composition studies prove the reactivity of quartz-glaukonitic sandstone with clay-carbonate cementing substance, which is mainly responsible for the reactivity of post-glacial gravel, containing this sandstone. Besides the obtained loss of mass, the occurrence of fine rock powder found at the bottom of beaker with NaOH solution, in which this sandstone was stored, can also indicate this. Before the measurements, all fractions with particle sizes lower than 8 mm were removed from the samples by rinsing in water; therefore, these residues observed after the test must have been formed during the measurement process. The degradation of sandstone occurring as a result of a reaction with sodium hydroxide is thus proved.

The observations under the microscope prove that this rock, whose mineralogical composition can be classified as intermediate between the sandstone and calcium gaize, is a polymineral material with various forms of reactive silica. Chalcedony-LF occurring in silicate and carbonate bioclasts can be regarded as such form. However, when the chalcedony content in sandstone composition lower than 3%, it is considered to be too minimal to cause the potential reactivity of aggregate [43]. 

During the microstructural analysis, it has been found that the surface of silicate bioclasts is more inhomogeneous. Their surface is characterized by the occurrence of numerous microcracks, which increase reactivity. Simultaneously, the rosette features, characteristic for reactive lepispheres were not observed [44]. 

Apart from chalcedony, the microcrystalline silica, which is present in carbonate cement, was also found in sandstone. Quartz in the fine-grained form, sometimes is also considered as a chalcedony [26]. 

The form of reactive silica, which could not be detected by the methods used, and which has high reactivity, is moganite [10,26,45]. Its occurrence in the sample examined is highly probable, because it usually occurs with chalcedony [26,45]. Significant reactivity of sandstone examined as determined by chemical method, may indicate its potential presence in this rock [32]. 

Thus, based on aforementioned studies, it was found that sandstone contains two potentially reactive phases (chalcedony and microcrystalline silica) which may be responsible for its significant reactivity, and furthermore there is also the possibility of the occurrence of a third very reactive phase (moganite). The clay minerals also can contribute to the aggregate degradation, similarly as in the case of alkali-carbonate reactions [30]. In the tested sandstone, the presence of glaukonite, a ferruginous version of illite, was identified. However, clay minerals from the illite group are not swellable and can't cause the aggregate degradation [26]. 

All potentially reactive phases are located inside the carbonate cementing substance. For this reason, if reactive silica phases do not occur on the surface of aggregate particle, the active alkalis from concrete pore solution must be not only able to migrate through the cement paste matrix, but also through the aggregate grain to reactive phases occurring in this aggregate. In the case of discussed sandstone, this process will be facilitated, because of its significant open porosity amounting 10%. 

The structure of reactive aggregate described above brings about that alkali-silica reaction will run in similar way as for limestone aggregates. This case was described by Monnin et al. [46]. Sandstone described by Rivard et al. [28], in which poorly crystallized siliceous cementing substance is the reactive phase bonding unreactive phases, behaves differently. 

The occurrence of cracked aggregate particles, which can be identify as quartz-glaukonitic sandstone, has been found during the analysis of mortars fractures under the scanning electron microscope. The cementing material in sandstone rich in calcium and the occurrence of specific, spherical silica agglomerations, which can be the bioclasts, indicate this. The degradation process of the aggregate with spherical cracks formation proceeded in the most visible way, just around these agglomerations. Apart from silicon, the sodium occurs in significant quantities in these agglomerations (in contrast to those observed in the rock). Their occurrence can be explained by chemical processes on the surface of these agglomerations during the alkali-silica reaction [3,24]. For this reason, they can be considered as reactive silica agglomerations coated with a layer of the alkaline silica gel [42].

In the testing mortar, the characteristic acicular forms were formed in microcracks between silica agglomerations and aggregate cement as a result of alkali-aggregate reaction. As part of the reaction, products of sodium ions with reactive silica spread on the mortar’s surface. They can be formed as a result of the silica gel crystallization responsible for the cracks formation and propagation.

From the viewpoint of concrete degradation mechanism as a result of alkali-aggregate reaction, calcium ions, which can react with alkaline silica gel, also play an important role, apart from sodium and potassium ions [24]. It has been discovered that in mortar, a thin layer rich in calcium occurs on the rock cementing substance around the silica agglomerations (Figure 9a,d), and it can be the source of these ions. Significant amounts of calcium (Figure 9b) found on the silica surface could stay on it after separation from the envelope, which surrounds it. However, due to the small thickness of this layer, it cannot be proven that the silica agglomerations are chalcedony filling the carbonate bioclasts, observed during the petrographic analysis of sandstone (Figure 3). 

The calcite cementing material of sandstone can be also the source of Ca^2+^ ions in an alkali-silica reaction. This happens because the so-called “common ion effect” can occur as a result of the presence of large quantity of OH^−^ ions derived from sodium and potassium hydroxides dissolved in a pore solution [47,48]. As a result of this effect, the solubility and content of Ca^2+^ ions in solution are decreased. Thus, the solubility of Ca(OH)_2_, which can transform into calcite, will be decreased. Ca^2+^ ions in this form can react with the alkaline silica gel formed by reaction of alkalis with reactive silica. 

Observations of the mortars under the scanning electron microscope do not reveal damages, which were formed through the reaction of alkalis with micro- and cryptocrystalline quartz from cementing substance, found during the analysis of polished sections of sandstone. It could result from the accumulation of gel formed in sandstone pores. Then this could hinder the access of alkalis to the other reactive phases by blocking the pores in sandstone.

## 5. Conclusions

As it can be derived from the data presented in this study there is a possibility of degradation of quartz-glaukonitic sandstone with clay-carbonate cementing material due to the impact of alkalis. The occurrence of chalcedony from bioclasts is responsible for this degradation.

Particles of aggregate can be more damaged due to the reaction with alkalis than the surrounding cement paste. The occurrence of expansion, which can contribute to concrete failure depends on the conditions of this reaction. Regardless of them, the degradation of sandstone particles differs from model alkali-aggregate reaction. It can also different from the degradation of other sandstones [28]. The tested aggregate can react with alkalis according to the following scheme:

1. Release of calcium, alkalis and OH^−^ ions to the sandstone particles through the pores and microcracks of cement paste;

2. Release of aforementioned ions through the sandstone pores to reactive silica agglomerations occurring in the form of bioclasts chalcedony (possibility of reaction with microcrystalline silica from cementing substance during this ions flow); 

3. Formation of swelling, alkaline silica gel, which is accompanied by cracks formation, especially around bioclasts;

4. Reaction of silica gel with Ca^2+^ ions coming from the solution, to which these ions can be released from aggregate cement;

5. Formation of well crystallized products of alkali-silica reaction in the formed cracks.

Thus, the migration of alkalis through the two different components (hardened paste and aggregate cementing substance) causes an additional complications of alkali-aggregate reaction process, which should be taken into account during consideration of natural aggregates reactivity. 

## Figures and Tables

**Table 1:** Gravel petrographic composition (%).

Quartz-Glaukonite Sandstone	Organo-Dendritic, Sparite-Micrite Limestone	Metamorphous Quartz-Pyroxene Shale with Opal	Feldspar-Biotite Granite	Unreactive Component (e.g., Limestone, Dolostone, Granite, Quartzite)
13.8	28.0	3.9	10.5	43.8

**Table 2:** Cement chemical composition (%).

Material	SiO_2_	Al_2_O_3_	Fe_2_O_3_	CaO	MgO	SO_3_	Alkali		TiO_2_	LOI	I.R.
							K_2_O	Na_2_O			
Cement	20.06	4.77	2.98	61.29	1.79	3.01	1.14	0.15	0.45	2.98	0.99

**Table 3:** Mineral composition of quartz-glaukonite sandstone.

Mineral Component	Content (%)
Cement	50.8
Quartz	25.4
Potassium feldspar	0.4
Plagioclase	0.2
Chalcedony-LF	7.2
Muscovite	0.6
Calcite	4.4
Glaukonite	11.0

**Table 4:** Results of sandstone open porosity and its bulk density.

Open Porosity (%)	Apparent Density (g·cm^−3^)
10	2.26
